# Evaluation of adherence to pharmacological treatment in a large sample of patients with personality disorder

**DOI:** 10.1192/j.eurpsy.2025.1911

**Published:** 2025-08-26

**Authors:** M. Cocchi, N. Girone, M. Leonardi, F. Achilli, B. Benatti, B. M. Dell’Osso

**Affiliations:** 1 Department of Mental Health, Department of Biomedical and Clinical Sciences Luigi Sacco, University of Milan; 2 Aldo Ravelli Center for Neurotechnology and Brain Therapeutic; 3 “Aldo Ravelli” Center for Neurotechnology and Brain Therapeutic, Milan, Italy; 4 Department of Psychiatry and Behavioral Sciences, Bipolar Disorders Clinic, Stanford University, Stanford, CA, United States

## Abstract

**Introduction:**

Personality disorders (PD) are defined as enduring and pervasive patterns of internal experience and behavior that deviate markedly from the expectations of the individual’s culture, causing distress and functional impairment (APA, 2013). Pharmacological therapy can be crucial to manage specific symptoms or psychiatric comorbidities, improving the quality of life of these patients. Adherence to pharmacological treatment is often reduced in patients with PD, representing a significant challenge for clinicians (Åkerblad et al., 2008). Few studies have explored this topic, highlighting the need for further research.

**Objectives:**

The aim of the present study was to assess medication adherence in patients with primary diagnosis or comorbidity of PD in different clinical settings of an Italian psychiatric department, considering clinical and socio-demographic differences.

**Methods:**

A sample of **200 patients diagnosed with PD** was recruited from various psychiatric services from the Department of Psychiatry at Luigi Sacco University Hospital, in Milan. Diagnoses were made through a structured clinical interview based on DSM-5 criteria (APA, 2013). The patient’s adherence to treatment was evaluated using the **Clinician Rating Scale (CRS)**, with a cut-off of ≥ 5 defining adherence subgroups [**Positive Adherence (A+): score ≥ 5; Negative Adherence (A-): score < 5].**

**Results:**

**Positive adherence** to pharmacological treatment was observed in **64.5%** of the sample, with significantly higher rates in patients with **Cluster C**(15.5% vs. 5.6%, p<0.005)(Figure 1). Furthermore, higher rates of positive adherence emerged in patients with a **positive family history** (60.3% vs 45.5%, p < 0.005). Finally, the analyzes between the different clusters revealed a significantly **higher lifetime prevalence of suicidal thoughts in Cluster B** (63.0%, p<0.05) and that the **majority of Cluster A** (60%, p<0.05) came **from territorial mental health services**, while the **majority of Cluster C** (66.7%, p<0.05) **from day hospital services** (Figure 2).

**Image 1:**

**Figure:**
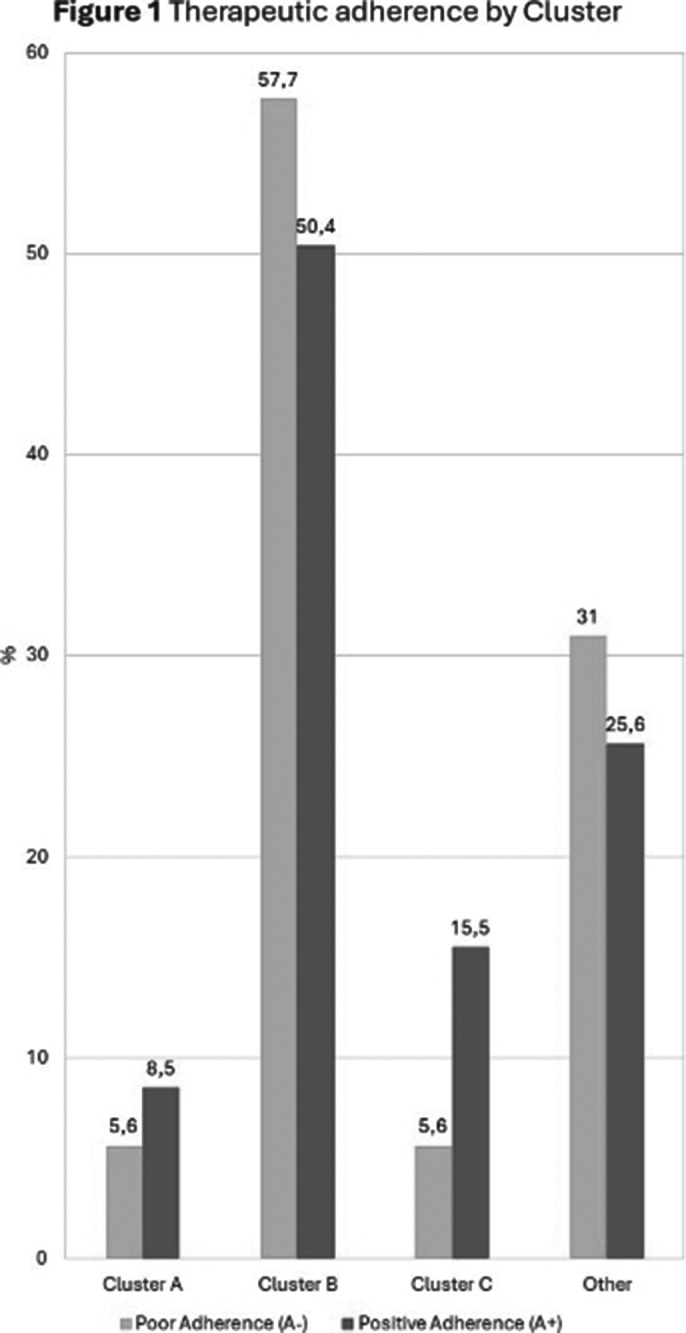

**Image 2:**

**Figure:**
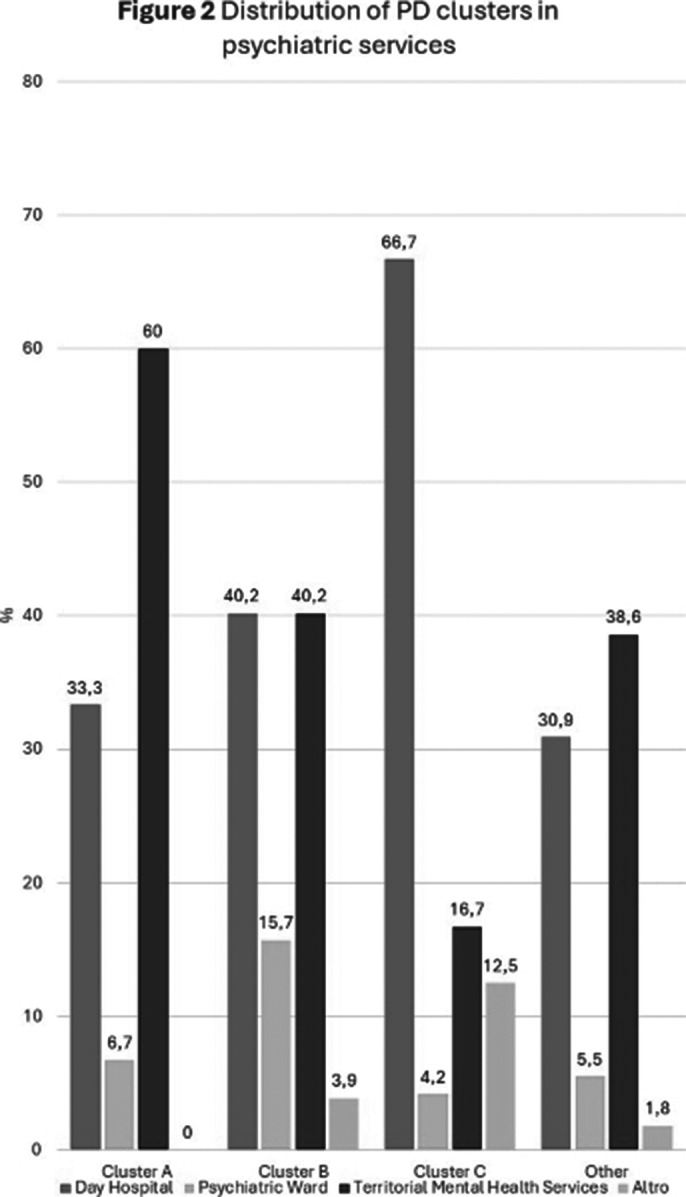

**Conclusions:**

The study found significantly higher treatment adherence rates in Cluster C patients, suggesting a **possible link between the anxious traits characteristic** of this group **and greater compliance**. Furthermore, a positive family history was associated with higher adherence rates, highlighting **the role of family influences in adherence**. Analyzing the differences between socio-demographic and clinical variables in the different clusters, it emerged that the prevalence of lifetime suicidal thoughts was significantly higher in Cluster B and that the patients in Cluster A mainly came from local mental health services and those in Cluster C from day hospital services. These findings highlight **the importance of personalized interventions to meet the specific needs** of each Cluster in order to optimize treatment adherence and therapeutic outcomes.

**Disclosure of Interest:**

None Declared

